# Dairy Consumption and the Risk of 15-Year Cardiovascular Disease Mortality in a Cohort of Older Australians

**DOI:** 10.3390/nu5020441

**Published:** 2013-02-06

**Authors:** Jimmy Chun Yu Louie, Victoria M. Flood, George Burlutsky, Anna M. Rangan, Timothy P. Gill, Paul Mitchell

**Affiliations:** 1 School of Health Sciences, Faculty of Health and Behavioural Sciences, The University of Wollongong, NSW 2522, Australia; E-Mail: jlouie@uow.edu.au; 2 Cluster for Public Health Nutrition, Boden Institute of Obesity, Nutrition, Exercise and Eating Disorders, The University of Sydney, NSW 2006, Australia; E-Mails: anna.rangan@sydney.edu.au (A.M.R.); t.gill@sydney.edu.au (T.P.G.); 3 Centre for Vision Research, Department of Ophthalmology, Westmead Millennium Institute, The University of Sydney, NSW 2006, Australia; E-Mails: george.burlutsky@sydney.edu.au (G.B.); paul.mitchell@sydney.edu.au (P.M.); 4 Discipline of Nutrition and Metabolism, School of Molecular Bioscience, The University of Sydney, NSW 2006, Australia

**Keywords:** dairy, cardiovascular disease, older Australian, Blue Mountains Eye Study, population

## Abstract

The effects of habitual dairy consumption and the risk of 15-year cardiovascular disease (CVD) mortality in a cohort of older Australians were investigated. Participants (*n* = 2900) completed a validated 145-item semi-quantitative food frequency questionnaire. Cox proportional hazards regression models were used to investigate associations between tertiles of the dairy consumption, including low/reduced fat dairy, whole fat dairy and their ratio (ratio_LF/WF_), and risk of mortality from coronary heart disease (CHD), stroke or combined CVD. There were 548 recorded cases of CVD mortality in this cohort. For total dairy intake, a reduction in risk of CVD was only seen in tertile 2 (adjusted hazard ratio, AHR: 0.71; 95% CI: 0.55–0.93), and for CHD both tertile 2 and tertile 3 were associated with a reduced risk (both with AHR: 0.71). However there were no linear trends between total dairy consumption and any of the three outcomes. There were no associations or trends between low/reduced fat dairy or whole fat dairy consumption, or ratio_LF/WF_ and any of the three outcomes in the fully adjusted model (all *p* > 0.05). This study found no consistent association between baseline consumption of dairy foods and the risk of CHD, stroke and combined CVD mortality.

## 1. Introduction

There is growing interest in the potential health benefits of dairy foods beyond bone health. Many studies have explored the health benefits offered by regular dairy consumption [1,2,3,4], with potential protective effects on the heart one of the most frequently investigated benefits [5,6,7].

Since dairy foods, particularly the whole fat varieties, are naturally high in saturated fat [8], intake of dairy foods was initially believed to increase the risk of cardiovascular disease (CVD). This was hypothesized because high saturated fat intake was shown to increase serum LDL cholesterol [9], a strong risk factor for CVD. Recommendations were therefore developed to limit or replace whole fat dairy products with low fat or skimmed varieties [10].

Prospective cohort studies [5,6,7,11] attempting to link dairy intake with risk of incident CVD, however, identified mixed outcomes. An earlier systematic review published in 2001 concluded that current clinical and biomedical evidence supports the hypothesis that dairy consumption may reduce the risk of stroke [12]. Another systematic review of 12 prospective cohort studies published in 2009 concluded that there was no consistent evidence associating dairy foods with the risk of coronary heart disease (CHD) [13]. A meta-analysis of 38 prospective cohort studies published in 2010 investigating dairy consumption found significant reductions in risk of ischemic heart disease and stroke for subjects with the highest dairy consumption [14]. However, another meta-analysis of 17 studies published in 2011 reported that milk intake was only modestly inversely associated with overall CVD risk, and there was no significant association between milk consumption and all-cause mortality, or risk of CHD or stroke [15]. Similarly, studies investigating the association between dairy consumption and CVD mortality have reported mixed results [6,13,16,17,18,19], with some studies reporting a small protective effect and others reporting no effect.

A previous study by our group [4] in a cohort of Australian aged 49 years or older (the Blue Mountain Eye Study; BMES) had shown that higher whole fat dairy intake was associated with reduced risk of metabolic syndrome but not type 2 diabetes, and higher low/reduced fat dairy intake was associated with an increased risk of metabolic syndrome. Because metabolic syndrome is a risk factor of CVD mortality, we aimed in this study to investigate the effect of habitual dairy consumption on the risk of 15-year mortality from CVD, including CHD and stroke using data from the BMES.

## 2. Methods

### 2.1. Study Population

The BMES is a population-based cohort study of vision, common eye diseases and other health outcomes in residents aged 49 years and over in the Blue Mountains area, west of Sydney, Australia [20]. During 1992–1994, of 4433 eligible residents aged 49–97 years, 3654 people attended detailed clinical examinations, and 3267 participants (89.4%) attempted and returned the baseline food frequency questionnaire (FFQ), of which 2900 were usable (79.4% of the participants examined). 

This study was conducted according to guidelines from the Declaration of Helsinki and all procedures involving human subjects/patients were approved by the Sydney West Area Health Service and University of Sydney Human Research Ethics Committees. Written, informed consent was obtained from all subjects. 

### 2.2. Dietary Assessment

Dietary data were collected using a 145-item self-administered semi-quantitative FFQ, modified for the Australian diet and vernacular from an early FFQ by Willett *et al.* [21]. Respondents were asked to indicate their usual frequency of consuming food items during the past year, using a nine-category frequency scale that ranged from never to four or more times per day. Each food was presented on the FFQ with a standard portion size. An allowance for seasonal variation of fruit and vegetables was made by weighting seasonal fruits and vegetables. Dietary intakes were reassessed by the same FFQ every 5 years. The FFQ has been tested for reproducibility and validity in a subsample of the study population against weighed food records for nutrients but not individual food items [22]. The Spearman correlation coefficient between FFQ and weighed food record for total calcium was 0.61 with less than 1% of the subjects grossly misclassified which indicates good validity. The Spearman correlation coefficient for long-term reproducibility for total calcium is 0.69 which indicates good reproducibility.

Nutrient intakes were estimated using the Australian Tables of Food Composition (NUTTAB90) [23] for baseline dietary data. Dairy sub-categorization includes whole fat milk, reduced fat/skim milk, low fat cheese, whole fat cheese, reduced fat dairy dessert (e.g., low fat yoghurt), and medium fat dairy dessert (e.g., custard and whole fat yoghurt). In Australia the term whole fat dairy products does not include high fat dairy products such as cream and butter. For the purpose of this analysis, total dairy included all of dairy foods; low/reduced fat dairy included “reduced fat/skim milk”, “reduced fat dairy dessert” and “low fat cheese”; while whole fat dairy included “whole fat milk”, “whole fat cheese” and “medium fat dairy dessert”. Tertiles of dairy product consumption were based on serves of dairy consumed per day. The serving sizes used were 250 mL for milk, 200 g for yoghurt, 250 mL for custards, and 40 g for cheeses based on the Australian Guide to Healthy Eating [10]. Buttermilk, flavored milk and cream cheese were not included in the FFQ items, but they were not expected to be consumed in large quantity by this population based on previous data [24]. A detailed list of the number of questions asked for all dairy categories, as well as food categories used in the analyses is available as Supplemental Table S1. 

### 2.3. Collection and Definition of Covariates

Weight and height of the participants were measured at each study visit. Physical activity was assessed using a standardized interviewer-administered questionnaire as previously described [20]. History of smoking was defined as “never”, “past”, or “currently smoking”, which included those who had stopped smoking within the past year. Blood pressure (BP) and fasting blood glucose level (FBG) were assessed during each visit. Stage II hypertension was defined as systolic BP ≥ 160 mmHg and/or diastolic BP ≥ 100 mmHg, or if subjects were previously diagnosed as hypertensive and were currently using anti-hypertensive medications. Type 2 diabetes mellitus (T2DM) was defined as self-reported diagnosis of T2DM and taking medication for T2DM, or FBG ≥ 7.0 mmol/L. Previous history of stroke, angina and acute myocardial infarction were defined using self-reported physician diagnosis. Participants self-reported their use of medications, and were instructed to bring all medications with them at each visit for study personnel to check.

### 2.4. Definitions of the Health Outcomes of Interest

CVD mortality data were obtained by matching the causes of death by CHD or stroke to the National Death Index (NDI), for approximately 95% of the participants up until 31 December 2007 (*i.e.*, 15 years of follow up). Those who were unable to be matched to the NDI were excluded from the analysis. Causes of death in the NDI were defined using the 9th revision of International Classification of Diseases Code (ICD-9) and International Statistical Classification of Diseases, 10th revision (ICD-10), with the following codes used for CHD: (ICD-9:410.0 to 410.9, 411.0 to 411.8, 412.0, 414.0 to 414.9 and ICD-10:I21.0 to I21.9, I22.0 to I22.9, I23.0 to I23.8, I24.0 to I24.9 and I25.0 to I25.9) or stroke: (ICD-9:430.0 to 438.9 and ICD-10:I60.0 to I69.9). The data from Australian NDI has been validated, and reported to be highly sensitive and specific for cardiovascular mortality (92.5% and 89.6%, respectively) [25]. The CVD cases combine the data for CHD and stroke, and there were 60 cases where CHD and stroke were both the primary cause of death.

### 2.5. Study Sample

The details of the data cleaning process have been reported elsewhere [26]. In brief subjects with excessive missing FFQ data (>10% questions unanswered or missing data for all questions relating to a particular food group), extreme nutrient values or extreme reported energy intake (<2500 kJ or >18000 kJ per day) were excluded. On the basis of the above criteria, we excluded 367 FFQs at baseline, leaving 2896 subjects with usable FFQ. For multivariate analyses with dairy intake variables as the exposure variable, an additional 238 subjects were excluded due to unidentifiable causes of death or missing baseline data for one or more of the following variables: BMI (41 missing values), smoking status (83 missing values), and history of stroke (13 missing values), myocardial infarction (15 missing values), or type 2 diabetes mellitus (125 missing values), reducing the number of subjects to 2662. For multivariate analyses with ratio_LF/WF_ as the exposure variable, a further 37 subjects were excluded because of zero whole fat dairy intake resulting in division of zero in the calculation of ratio_LF/WF_. 

### 2.6. Statistical Analyses

All statistical analyses were performed using Statistical Analysis System version 9.1 (SAS Institute, Cary, NC). Cox proportional hazards regression models were used to investigate the associations between tertiles of total, whole fat and low/reduced fat dairy consumption and the ratio of low/reduced fat dairy to whole fat dairy (ratio_LF/WF_) at baseline, and risk of mortality from CHD, stroke or CVD. Risk estimates were adjusted for known risk factors for cardiovascular mortality. The base model included adjustment for age and sex. Further adjustment for total energy intake, body mass index (BMI), change in weight during follow up, previous acute myocardial infarction, previous stroke, smoking status, stage II hypertension at baseline and type 2 diabetes status at baseline, use of hypertensive medications and statins, and change in dairy intake were made to Model 1, which was used as the fully adjusted model. Change in dairy intake was calculated as dairy intake reported at second study visit less that at first study visit; if the subject died before the second study visit, change in dairy intake was set as 0; for analyses of total dairy, change in total dairy intake was included in the model; for analyses of low/reduced fat dairy and whole fat dairy, change in both types of dairy were included in the model; for analysis of ratio_LF/WF_, change in ratio_LF/WF_ was included in the model. Collinearity between the dairy intake variables and the change in dairy intake variables were examined using the variance inflation factor (VIF) test and all of the VIF were found to be below 1.5, which is much lower than the acceptable limit of 10, indicating a low possibility of collinearity. Other dietary parameters (including intake of fish, fruit and vegetables) and socioeconomic status were not included in the final model as these were not significant in any of the models tested. There was no interaction between BMI and dairy intake and hence we did not stratify the analyses by BMI.

## 3. Results

The mean age of the study population at baseline was 65.4 (±SD 9.3) years. During the 15-year follow up, 1048 cases of all-cause mortality were recorded, of which 432 cases were attributable to CHD, and 176 to stroke and 548 to combined CVD. The mean daily total dairy intake of the study population was 1.7 (±SD 1.2) serves, which includes 1.1 (±SD 1.0) serves of milk, 0.4 (±SD 0.5) serves of cheese and 0.1 (±SD 0.3) serves of yoghurt. Due to the low reported intake of cheese and yoghurt, we were unable to undertake a separate analysis on the individual dairy food types. There were 0.4% non-consumers for total dairy, 1.4% non-consumers for full fat dairy, and 27.6% non-consumers for low/reduced fat dairy in the study population, and no subjects in T2 and T3 had zero intakes or ratio_LF/WF_. Table 1 shows the characteristics of the participants at baseline by tertiles of dairy intake. Participants who had higher total dairy intake had higher energy, calcium, sodium, total fat and saturated fat intake (all *P*_trend_ < 0.001). Similar trends existed for tertiles of whole fat dairy consumption and there were more smokers (*P*_trend_ = 0.006) and male participants in the higher tertiles (*P*_trend_ < 0.001). For low/reduced fat dairy, there were less male participants (*P*_trend_ < 0.001) and smokers (*P*_trend_ < 0.001) in the higher tertiles. Those who had more low/reduced fat dairy also tended to be younger (*P* < 0.001), had higher BMI, energy intake and calcium intake (all *P*_trend_ < 0.001), but had lower intakes of total fat and saturated fat (all *P*_trend_ < 0.001).

**Table 1:** Subject characteristics at baseline by tertiles of dairy intake. Values are mean ± SD except for male and smoker, which were given as percentages. * *p* for trend, except for categorical variables which were tested by Pearson’s χ^2^. BMI, Body Mass Index; AMI, acute myocardial infarction; Ratio_LF/WF_, Ratio between low fat dairy and whole fat dairy.

		T1	T2	T3	*p* value *
**Total dairy**					
	Age (year)	65.5 ± 9.4	65.3 ± 9.4	65.2 ± 9.1	0.760
	Male (%)	47.0	43.7	41.5	0.053
	Smokers (%)	15.8	12.9	13.5	0.168
	BMI (kg/m^2^)	26.0 ± 4.6	26.1 ± 4.4	26.4 ± 4.3	0.231
	Energy (kJ)	7340.7 ± 2324.7	8449.0 ± 2280.0	9780.7 ± 2518.0	<0.001
	Calcium (mg)	517.4 ± 172.2	809.8 ± 158.3	1345.4 ± 338.0	<0.001
	Total fat (g)	64.2 ± 25.5	75.3 ± 24.9	88.6 ± 31.2	<0.001
	Saturated fat (g)	23.8 ± 11.0	29.3 ± 11.1	36.4 ± 15.0	<0.001
	Fish intake (g)	25.6 ± 27.1	27.1 ± 28.0	29.6 ± 2.79	0.005
	Fruit intake (g)	303.8 ± 283.7	329.1 ± 230.2	395.0 ± 260.6	<0.001
	Vegetable intake (g)	408.2 ± 200.9	439.2 ± 180.7	477.7 ± 197.8	<0.001
	Previous AMI (%)	8.3	8.4	8.7	0.952
	Previous stroke (%)	4.7	4.7	4.8	0.990
	Previous angina (%)	11.5	11.6	11.8	0.982
	Type 2 diabetes (%)	5.5	7.8	9.3	0.007
	Stage II hypertension (%)	47.5	45.2	43.2	0.165
	Using anti-hypertensive drug (%)	43.1	41.9	38.1	0.646
	Using statins (%)	3.7	3.0	3.3	0.674
	Education level: degree or above (%)	57.4	60.0	61.9	0.147
**Low/reduced fat dairy**					
	Age (year)	66.8 ± 9.9	64.3 ± 9.3	65.1 ± 8.6	<0.001
	Male (%)	54.3	43.1	35.3	<0.001
	Smokers (%)	19.1	14.3	9.2	<0.001
	BMI (kg/m^2^)	25.6 ± 4.3	26.5 ± 4.5	26.3 ± 4.4	<0.001
	Energy (kJ)	8599.0 ± 2658.3	8224.0 ± 2654.5	8744.2 ± 2389.8	<0.001
	Calcium (mg)	746.0 ± 360.0	755.7 ± 326.8	1158.1 ± 414.5	<0.001
	Total fat (g)	80.6 ± 29.6	74.9 ± 30.3	72.8 ± 26.8	<0.001
	Saturated fat (g)	33.1 ± 14.2	29.1 ± 13.6	27.4 ± 12.1	<0.001
	Fish intake (g)	23.6 ± 26.7	27.8 ± 26.6	30.7 ± 29.2	<0.001
	Fruit intake (g)	293.2 ± 260.6	341.8 ± 252.1	389.8 ± 263.9	<0.001
	Vegetable intake (g)	423.3 ± 202.2	437.9 ± 195.3	462.8 ± 186.8	<0.001
	Previous AMI (%)	6.5	7.9	10.9	0.001
	Previous stroke (%)	5.5	3.5	5.1	0.096
	Previous angina (%)	9.0	11.1	14.7	<0.001
	Type 2 diabetes (%)	6.5	7.7	8.4	0.292
	Stage II hypertension (%)	45.7	44.8	45.4	0.925
	Using anti-hypertensive drug (%)	40.9	38.7	43.5	0.098
	Using statins (%)	1.4	3.3	5.2	<0.001
	Education level: degree or above (%)	54.7	64.3	59.8	0.002
**Whole fat dairy**					
	Age (year)	65.0 ± 8.8	65.4 ± 9.2	65.7 ± 9.9	0.327
	Male (%)	39.3	43.8	49.2	<0.001
	Smokers (%)	14.4	11.3	16.5	0.006
	BMI (kg/m^2^)	26.3 ± 4.5	26.1 ± 4.5	26.2 ± 4.4	0.706
	Energy (kJ)	7460.7 ± 2198.7	8377.2 ± 2404.6	9742.3 ± 2582.2	<0.001
	Calcium (mg)	731.1 ± 383.2	835.3 ± 373.1	1106.9 ± 399.7	<0.001
	Total fat (g)	60.4 ± 23.0	73.9 ± 25.0	94.0 ± 28.5	<0.001
	Saturated fat (g)	21.3 ± 9.6	28.4 ± 10.6	39.8 ± 13.0	<0.001
	Fish intake (g)	28.5 ± 30.3	27.7 ± 26.0	26.1 ± 26.5	0.150
	Fruit intake (g)	340.3 ± 280.2	337.1 ± 247.0	350.3 ± 257.0	0.513
	Vegetable intake (g)	420.3 ± 190.3	443.7 ± 191.9	461.4 ± 201.7	<0.001
	Previous AMI (%)	11.8	7.2	6.4	<0.001
	Previous stroke (%)	5.5	4.0	4.6	0.287
	Previous angina (%)	14.9	10.9	9.1	<0.001
	Type 2 diabetes (%)	7.4	6.0	9.2	0.034
	Stage II hypertension (%)	50.0	43.3	42.5	0.001
	Using anti-hypertensive drug (%)	48.6	38.5	35.9	<0.001
	Using statins (%)	5.6	3.0	1.3	<0.001
	Education level: degree or above (%)	58.0	59.9	38.9	0.393
**Ratio_LF/WF_**					
	Age (year)	65.6 ± 9.9	64.5 ± 9.3	65.0 ± 8.7	0.327
	Male (%)	54.0	42.5	35.8	<0.001
	Smokers (%)	19.3	13.2	10.2	<0.001
	BMI (kg/m^2^)	25.6 ± 4.2	26.6 ± 4.6	26.3 ± 4.3	0.706
	Energy (kJ)	8791.7 ± 2642.3	8672.2 ± 2746.2	8191.9 ± 2266.6	<0.001
	Calcium (mg)	797.2 ± 370.2	874.6 ± 432.8	1007.1 ± 410.8	<0.001
	Total fat (g)	83.1 ± 29.9	79.9 ± 30.2	66.2 ± 23.5	<0.001
	Saturated fat (g)	34.5 ± 14.3	31.5 ± 13.4	24.0 ± 10.1	<0.001
	Fish intake (g)	23.8 ± 25.8	28.0 ± 26.7	29.8 ± 26.7	0.150
	Fruit intake (g)	298.5 ± 257.7	360.6 ± 263.0	371.1 ± 260.8	0.513
	Vegetable intake (g)	425.5 ± 196.3	455.0 ± 203.7	447.0 ± 182.5	<0.001
	Previous AMI (%)	6.0	7.0	12.0	<0.001
	Previous stroke (%)	5.4	3.6	5.3	0.119
	Previous angina (%)	8.6	9.9	16.0	<0.001
	Type 2 diabetes (%)	6.8	8.4	7.2	0.417
	Stage II hypertension (%)	45.3	42.8	47.5	0.112
	Using anti-hypertensive drug (%)	40.7	36.1	46.2	<0.001
	Using statins (%)	13.8	26.6	59.6	<0.001
	Education level: degree or above (%)	54.3	64.8	60.0	<0.001

**Table 2:** Hazard Ratio ^†^ (95% Confidence Interval) of cardiovascular disease mortality according to tertiles of dairy intake. CVD, cardiovascular disease; IQR, interquartile range; Ratio_LF/WF_, ratio between low fat dairy and whole fat dairy. ^#^ The serving sizes used were 250 mL for milk, 200 g for yoghurt, 250 mL for custards, and 40 g for cheeses; ^†^ Hazard ratios calculated by Cox proportion hazard regression; ^‡^ Base model adjusted for age and sex; ^§^ Model 1: Base model with additional adjustment for total energy, baseline BMI, change in weight during follow up (kg), physical activity level (METs), previous acute myocardial infarction (yes/no), previous stroke (yes/no), smoking status (yes/no), stage II hypertension (yes/no), type 2 diabetes status (yes/no), use of antihypertensive medication (yes/no), use of statins (yes/no) and change in dairy intake; * *p* < 0.05 when compared to tertile 1.

		*n*	Median (IQR)	Total CVD			Stroke			Coronary Heart Diseases		
				Deaths	Base model ^‡^	Model 1 ^§^	Deaths	Base model ^‡^	Model 1 ^§^	Deaths	Base model ^‡^	Model 1 ^§^
**Total dairy (serves/day) ^#^**	**T1 **	965 ^‡^	0.6	197 ^‡^	1.00	1.00	56 ^‡^	1.00	1.00	159 ^‡^	1.00	1.00
	**(ref)**	879 ^§^	(0.3–0.9)	176 ^§^			50 ^§^			143 ^§^		
	**T2**	967 ^‡^	1.4	175 ^‡^	0.81 *	0.71 *	63 ^‡^	0.99	0.86	136 ^‡^	0.78 *	0.71 *
		883 ^§^	(1.2–1.6)	158 ^§^	(0.66–0.99)	(0.55–0.93)	58 ^§^	(0.69–1.42)	(0.53–1.40)	123 ^§^	(0.62–0.99)	(0.53–0.96)
	**T3**	964 ^‡^	2.9	176 ^‡^	0.90	0.76	57 ^‡^	1.01	0.98	137 ^‡^	0.87	0.71 *
		900 ^§^	(2.4–3.3)	157 ^§^	(0.73–1.10)	(0.56–1.02)	50 ^§^	(0.70–1.46)	(0.58–1.16)	122 ^§^	(0.69–1.09)	(0.51–0.99)
	***p*_trend_**	-	-	-	0.762	0.500	-	0.464	0.775	-	0.974	0.318
**Low/reduced fat dairy (serves/day)**	**T1 **	934 ^‡^	0.0	222 ^‡^	1.00	1.00	75 ^‡^	1.00	1.00	172 ^‡^	1.00	1.00
	**(ref)**	841 ^§^	(0.0–0.0)	192 ^§^			64 ^§^			149 ^§^		
	**T2**	968 ^‡^	0.1	155 ^‡^	0.81 *	0.89	49 ^‡^	0.76	1.00	120 ^‡^	0.80	0.84
		892 ^§^	(0.1–0.5)	143 ^§^	(0.65–0.99)	(0.68–1.05)	45 ^§^	(0.53–1.09)	(0.63–1.59)	112 ^§^	(0.63–1.01)	(0.62–1.13)
	**T3**	994 ^‡^	1.2	171 ^‡^	0.87	0.80	52 ^‡^	0.78	0.84	140 ^‡^	0.92	0.87
		929 ^§^	(1.0–2.5)	156 ^§^	(0.71–1.07)	(0.61–1.06)	49 ^§^	(0.54–1.13)	(0.52–1.37)	127 ^§^	(0.73–1.16)	(0.64–1.18)
	***p*_trend_**	-	-	-	0.756	0.205	-	0.777	0.434	-	0.916	0.659
**Whole fat dairy (serves/day)**	**T1 **	977 ^‡^	0.1	179 ^‡^	1.00	1.00	53 ^‡^	1.00	1.00	146 ^‡^	1.00	1.00
	**(ref)**	901 ^§^	(0.1–0.3)	164 ^§^			49 ^§^			133 ^§^		
	**T2**	952 ^‡^	0.8	177 ^‡^	0.95	1.03	52 ^‡^	0.94	1.02	146 ^‡^	0.96	1.03
		883 ^§^	(0.5–0.8)	162 ^§^	(0.77–1.17)	(0.80–1.34)	49 ^§^	(0.64–1.38)	(0.64–1.64)	134 ^§^	(0.77–1.21)	(0.77–1.37)
	**T3**	967 ^‡^	1.8	192 ^‡^	0.92	0.86	71 ^‡^	1.13	1.01	140 ^‡^	0.83	0.77
		878 ^§^	(1.3–2.6)	165 ^§^	(0.75–1.13)	(0.64–1.16)	60 ^§^	(0.79–1.62)	(0.64–1.64)	121 ^§^	(0.66–1.05)	(0.55–1.08)
	***p*_trend_**	-	-	-	0.809	0.362	-	0.065	0.980	-	0.170	0.148
**Ratio_LF/WF_**	**T1 **	952 ^‡^	0.0	220 ^‡^	1.00	1.00	74 ^‡^	1.00	1.00	170 ^‡^	1.00	1.00
	**(ref)**	856 ^§^	(0.0–0.0)	192 ^§^			64 ^§^			149 ^§^		
	**T2**	952 ^‡^	0.3	160 ^‡^	0.84	0.88	56 ^‡^	0.87	1.08	120 ^‡^	0.81	0.83
		881 ^§^	(0.1–0.8)	145 ^§^	(0.68–1.03)	(0.63–1.24)	51 ^§^	(0.61–1.23)	(0.60–1.96)	109 ^§^	(0.64–1.03)	(0.57–1.22)
	**T3**	952 ^‡^	5.6	161 ^‡^	0.85	0.94	42 ^‡^	0.65 *	0.81	137 ^‡^	0.94	1.04
		888 ^§^	(2.9–12.0)	148 ^§^	(0.69–1.05)	(0.68–1.31)	39 ^§^	(0.44–0.96)	(0.44–1.49)	126 ^§^	(0.75–1.18)	(0.72–1.49)
	***p*_trend_**	-	-	-	0.589	0.396	-	0.130	0.255	-	0.814	0.993

Table 2 shows the hazard ratios of combined CVD, stroke and CHD mortality according to tertiles of baseline dairy intake. Subjects whose total dairy intake was in tertile 2 had a 29% decreased risk of total CVD mortality in the fully adjusted model (multivariate adjusted HR: 0.71; 95% CI: 0.55–0.93). When split into subtypes of CVD, higher total dairy intake was found to be associated with a lower risk of CHD but not stroke. The reduction in CHD risk appeared to be equal (29% decreased risk) for those whose dairy intake was in tertiles 2 and 3. There were no significant linear trends between total dairy consumption and any of the three outcomes in both the base model and the fully adjusted model. We also examined non-linear trend for total CVD mortality across total dairy tertile and found there was no significant U-shaped relationship between total dairy intake and total CVD mortality (*p* = 0.080 in Model 1 with dairy intake entered as a quadratic term). No associations between low/reduced fat or whole fat dairy consumption and ratio_LF/WF_ and any of the three outcomes were seen in the fully adjusted model. Excluding prevalent CVD cases at baseline did not affect the results. Because of the high proportion of non-consumers for low/reduced fat dairy, we have conducted an additional analysis using the combined T1 and T2 for low/reduced fat dairy as a referent group, and found the HR for T3 to be similar (data not shown).

## 4. Discussion

Although the association between habitual dairy consumption and the risk of CVD mortality had been studied previously by many other groups [14,15,27], these results have been inconsistent. The present study is one of only few studies investigating such associations in an older population. We found that in a population-based cohort of older Australians, those with a medium intake (middle tertile) of total dairy had a significantly reduced risk of combined CVD mortality, and those with medium to high intakes (middle and upper tertiles) had a reduced risk of CHD mortality. It is however difficult to interpret these findings in the middle tertile and to determine whether it represents a true protective effect or are chance findings. There were no significant trends evident between increasing habitual dairy consumption (regardless of type) and the risk of CHD, stroke or combined CVD mortality. Nonetheless, there was no indication that increased dairy intake, even whole fat dairy, was associated with any increased risk of CVD in older subjects.

The Australian Guide to Healthy Eating (AGHE) recommends the consumption of 2 to 3 serves of dairy products per day to achieve a nutritionally adequate diet [10]. The study population reported a mean total dairy intake of 1.7 serves per day, which is less than the AGHE recommendation. This is comparable to the Australian national consumption data [24] and that reported by Bonthuis *et al.* [27] among Australian adults, despite the difference in dietary assessment method, comparing the FFQ to 24 h recall data. Bonthuis *et al.* [27] however reported different findings to the present study, where a strong protective effect was found for 16 year CVD mortality, but only in whole fat dairy (69% decreased risk for tertile 3 *vs.* tertile 1; *P*_trend_ = 0.04). Subjects in that study, however, had a much wider age range (25–78 years) than those of our study, which could explain the discrepancies, as the effect of dairy consumption and the pattern of dairy intake could differ between younger and older populations.

Previous studies suggested that dairy consumption was protective for incident stroke. In the Caerphilly cohort study [7], 665 men aged 45–59 years were followed for 20 years. The authors reported that participants whose daily milk intake was at or above the median intake (187 mL) were 48% (95% CI: 1%–73%; *P* = 0.05) less likely to experience a stroke incident. The study by Abbott *et al.* [28] also showed a reduction in rate of stroke with increasing milk intake (*P* < 0.05)—compared to non-milk drinkers, subjects who consumed at least 16 oz (448 mL) of milk per day were more than two times less likely to experience stroke (7.9% *vs.* 3.7%). The Alpha-Tocopherol, Beta-Carotene Cancer Prevention Study [29], on the other hand, showed no association between total dairy, low fat milk or regular milk consumption and the risk of most types of stroke, except for subjects who had the highest regular milk consumption (median intake 850 g/day), where a 41% increased risk (*P* < 0.05) of contracting intracerebral hemorrhage was found.

There is also evidence to support the protective effect of dairy calcium intake on stroke mortality. The Japan Public Health Center (JPHC) study [30] showed that, compared to those with the lowest dairy calcium intake (median = 0 mg/day), individuals who had the highest dairy calcium intake (median = 116 mg/day) had a 30% reduced risk for mortality from both total stroke and ischemic stroke (both *P* < 0.05). Similar results were reported by the same group earlier using data from the Japan Collaborative Cohort (JACC) study [31], which reported a 47% reduction in risk of mortality from both total stroke and ischemic stroke (both *P* < 0.05) among those people who had the highest dairy calcium intake (median = 127 mg/day). That study also reported a 27% (*P* < 0.05) reduced risk of mortality from combined CVD for those in the highest quintile of dairy calcium intake compared to those in the lowest quintile.

Evidence from studies investigating the association between dairy consumption and CVD mortality, however, is inconsistent. Three studies [16,17,18] reported no consistent association between dietary or dairy calcium intake and the risk of IHD mortality. Ness *et al.* [6] found no association between self reported daily milk intake at baseline and the risk of CHD, stroke or combined CVD mortality among 5765 men aged 35–64 years at baseline. The Rotterdam study [32] reported similar findings in 4664 adults aged ≥55 years. Similarly, the present study did not found significant trends between habitual dairy consumption and risk of CHD, stroke or combined CVD mortality. 

Studies that examined the effect of individual dairy products or ratio between whole fat and reduced fat dairy have produced different results. In the study by Mann *et al.* [33], while they found no association between the risk of IHD mortality and daily intake of milk, there was a significantly increasing trend in risk for higher cheese consumption. Similarly, while Hu *et al.* [34] found no association between either low/reduced fat or high fat dairy intake and the risk of CHD mortality in 80,082 women aged 34–59 years, they found a significant increasing trend in risk of CHD for increasing ratio between high fat and low/reduced fat dairy intake. We however did not find significant associations between ratio_LF/WF_ (an inverse ratio of what was used in the study by Hu *et al.* [34]) and risk of CVD mortality. 

In the same cohort [4] our group had previously shown that higher whole fat dairy intake was associated with reduced risk of metabolic syndrome but not type 2 diabetes, and higher low/reduced fat dairy intake was associated with an increased risk of metabolic syndrome. This is in contrast to the findings of the present study where higher intakes of total or low/reduced fat dairy appeared to be associated with a reduced risk of CVD mortality. The discrepancy of the finding could be due to the higher specificity of CVD mortality as an outcome as compared to that of metabolic syndrome, which is defined as a cluster of metabolic risk factors for CVD [35]. Subjects who met the criteria of metabolic syndrome do not necessarily develop CVD in the future—by definition they are only at a higher risk of developing CVD. These subjects may have been advised on dietary/lifestyle changes to reduce their risk of CVD. On the other hand, the time it takes for individuals at risk of CVD to encounter a fatal CVD event also varies. Therefore it may be interesting to follow-up this cohort for a longer period of time.

The strengths of the present study include the use of a validated FFQ with a high correlation coefficient for calcium to assess the dairy intake of the participants. This is likely to increase the plausibility of the associations found. The long follow up period also allowed us to better explore the temporal relationship between dairy consumption and CVD mortality. In addition, the application of the International Classification of Diseases Code results in accurate classification of the causes of death [25]. Furthermore, while this cohort was set up to study risk factors for vision diseases, this sample population is representative of the elderly Australian population except that the subjects had a higher SES.

However, the present study was limited in several ways. We have not presented data on non-fatal CVD as these were collected only via self-report, which had a high likelihood of report bias due to the potential cognitive and memory impairment of the older population. In addition, subjects with a CVD event are more likely to switch to a healthier diet, including the use of low/reduced fat dairy products, although this was adjusted for in the model. While using more than one FFQ collected more frequently (e.g., annually) may allow better detection in change in dairy consumption pattern, this would have imposed a significant burden on the study participants, which could have affected the response rate, and so was not adopted in the present study. We were also unable to rule out the effect of residual confounding.

## 5. Conclusions

There was no consistent association between baseline consumption of dairy foods and the risk of coronary heart disease, stroke and combined cardiovascular disease mortality.

## Supplementary Files

Supplementary File 1 Supplemental Table (PDF, 77 KB)
