# Supplementary material for: Dairy Consumption and the Risk of 15-Year Cardiovascular Disease Mortality in a Cohort of Older Australians
Source: Nutrients. 2013 Feb 6;5(2):441–54. doi: 10.3390/nu5020441 (PMC3635204; doi:10.3390/nu5020441)
Supplement: Supplementary File 1 — Supplemental Table (PDF, 77 KB) [file nutrients-05-00441-s001.pdf]

Supplemental Table

Table S1. Food categories used.

Major Food Group	Minor Food Group	Foods included	FFQ item ID
Vegetables			
	Potatoes		
	Low Fat	Boiled,mashed,baked	46,47
	High fat	Hot chips	48
	Brassica	Cabbage, cauliflower, broccoli and Brussel sprouts, coleslaw	54, 43, 42, 55, 62
	Leaf and stalk	Bean sprouts, celery, lettuce and silverbeet	64, 63, 61, 44
	Peas and Beans		52, 53, 50
	Other fruiting vegetables	Squash, zucchini, eggplant and pumpkin	58, 49
	Carrot and root		56, 51
	Tomatoes		60
	Legumes	Baked beans, soy, other	67, 66, 65
	Other vegetables	Mushrooms, corn and shallots	59, 57, 45, 141, 140
	Avocado		31
Cereal and cereal products			
	Breakfast cereals		91, 92
	Bread, white		93
	Bread, mixed grain and wholemeal		94
	Rice and pasta		96, 97, 98
Cereal-based products and dishes			
	Sweet biscuits	Sweet biscuits	130, 131
	Savoury biscuits	Crackers	99
	Cakes, buns, scones	Scone, pancakes, cakes, bun	95, 126, 129
	Pastries	Sweet pie	127, 128
	Mixed dishes	Pizza	139
Fruit			
	Citrus fruit	Orange, grapefruit	33, 34
	Tropical fruit	Pawpaw, pineapple, mango, banana	27, 28, 29, 35
	Pomme fruit	Pear, apple	32

Table S1. *Cont.*

	Berry fruit	Strawberry, raspberry, blueberry	24, 25
	Stone fruit	Peach, plum, apricot, nectarine	22
	Dried fruit	Prune, apricot, sultana, raisin, peach	36, 37, 38, 39
	Canned fruit	Fruit salad, apricot, peach, canned	40, 41
	Other fruit[e]	Rockmelon, watermelon, grapes	23, 26, 30
Non-alcoholic beverages			
	Tea		117, 118
	Coffee		115, 116
	Juices		104, 105, 106, 107, 108, 109
	Cordials		114
	Soft drinks		112, 113
	Artificially sweetened drinks	Low calorie soft drink	110, 111
Dairy Products			
Low/reduced fat dairy	Milk, reduced fat and low fat		6, 7
	Reduced fat dairy dessert	Low fat yoghurt	12
	Cheese, low fat	Cottage and ricotta cheese	13
Regular fat dairy	Milk, regular		8
	Medium fat dairy dessert	Custards and yoghurts	11, 125
	Cheese, regular	Cheddar chees	14
Meat			
Red Meat	Moderately-lean red meat cuts	Beef/pork/lamb trimmed	468
	Moderately-lean red meat dishes	Lamb stew, Beef stew, Hamburger patty, Beef rissole	69, 69, 74,
	Medium fat red meat cuts	Beef/pork/lamb partly trimmed	368
	Medium fat red meat dishes	Bolognaise sauce, Mince	75, 76
	High fat red meat cuts	Beef/pork/lamb not trimmed	268
	High fat red meat dishes	Sausages, meat pie, sausage roll	73, 79, 80

Table S1. *Cont.*

Processed meat		Ham, bacon, luncheon meat, frankfurter	70, 77, 81, 82
Organ meat		Liver	78,
Chicken	Moderately-lean chicken		72
	High fat chicken		71
Eggs	Low fat eggs	Egg, poached, hard-boiled	83
	Moderately fat eggs	Egg, scrambled, omelette, fried	84, 85
Fish	Seafood	Prawn, scallop, crab	90
	Low fat canned fish	Salmon, tuna in brine	87
	Moderately fat canned fish	Tuna, sardine in oil	86, 88
	Medium fat fish	Fish, other types	89
Nuts		Peanut, pecan, walnut, almond, peanut butter	135, 137
Fats			
	Butter		16
	Margarine		15
	Oils		160,161
	Cooking fat		162
Yeast/Beef extracts		Vegemite, marmite	136
Alcoholic beverages	Beer	Regular and reduced alcohol beer	119, 120
	Wine	Red, white and fortified wines	121, 122, 123
	Spirits		124
Confectionary		Lollies, chocolate	132, 133
Sugar products and dishes		Sugar, honey, jams, syrups	134, 156
Snack foods		Chips	138
Savoury sauces		French dressing, coleslaw dressing, mayonnaise	142, 143
